# Reimplantation of a Large Extruded Segment of Tibia in an Open Fracture in a Pediatric Patient

**DOI:** 10.1055/s-0039-1688952

**Published:** 2019-06-20

**Authors:** Ahmadreza Afshar, Ali Tabrizi

**Affiliations:** 1 Clinical Research Development Unit of Imam Khomeini Hospital, Urmia University of Medical Sciences, Urmia, Iran

**Keywords:** extruded bone, open fracture, reimplantation, tibia fracture, chlorhexidine gluconate, povidone–iodine

## Abstract

A 6-year-old boy presented with a Gustilo type IIIB open fracture on his left leg with a segment of bone loss in his tibia. The boy's attendants brought two bone fragments recovered from the scene of the accident. The extruded bones were a segment with a length of 5.5 cm and a cortical bone with a length of 4 cm. The extruded fragments were reimplanted after scrubbing with 10% povidone–iodine for 20 minutes, soaking in 2% chlorhexidine solution for 20 minutes, and rinsing with normal saline. Four months after the injury, the extruded fragments were incorporated in the callus of the fracture site and complete fracture union in appropriate alignment was achieved.

Extruded bone is a rare complication due to high energy in an open fracture. There are many challenges in the treatment of bone defect in open fractures. 1 The available information about the management of extruded bone is very limited. 1 2 There is an elevated risk of infection in the reimplantation of the extruded, contaminated, and devascularized bone segment. However, several case reports have described successful reimplantation of an extruded bone segment after cleaning with special techniques. 1 2 In all of these reports, the sterilization was conducted in different ways. Our knowledge of bone segment reimplantation is very limited. In this case report, we describe successful reimplantation of a large extruded segment of the tibia in an open fracture in a 6-year-old boy.

## Case Presentation

**Fig. 1:**
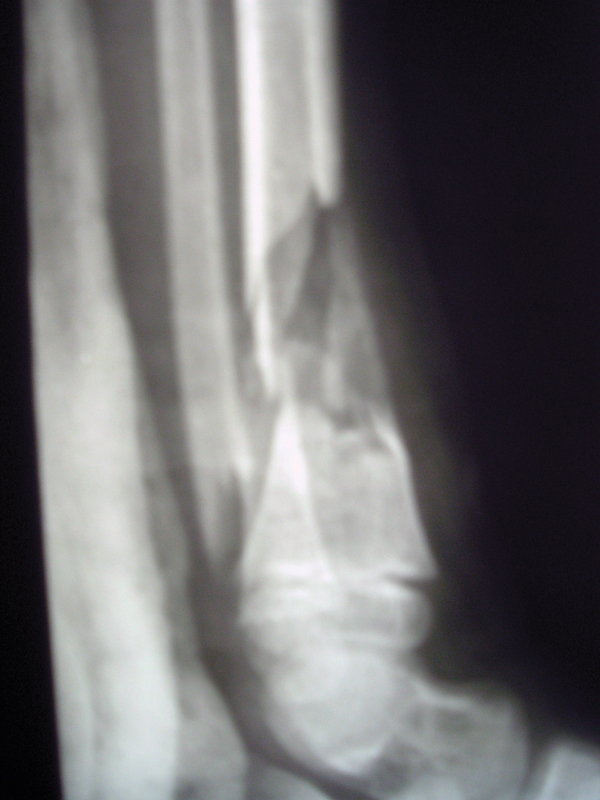
Lateral roentgenogram of the left leg demonstrates the bone defect of the distal tibia.

A 6-year-old boy presented with a Gustilo type IIIB open fracture with a segmental bone loss in his left tibia. His left leg was struck by the tire of an automobile. The boy's attendants brought two bone fragments recovered from the scene of the accident. The extruded bones were a segment with a length of 5.5 cm and a cortical bone with a length of 4 cm. The periosteum was stripped from the fragments, but the neurovascular structures of the left leg were intact ( Fig. 1 ).

The bone fragments were structurally important. In the operating room, a thorough and meticulous irrigation and a debridement of the bone and the soft tissues of the open tibia fracture were performed in accordance with the principles of open fracture management. There were a well-preserved periosteum and soft tissue envelope around the bone defect.

**Fig. 2:**
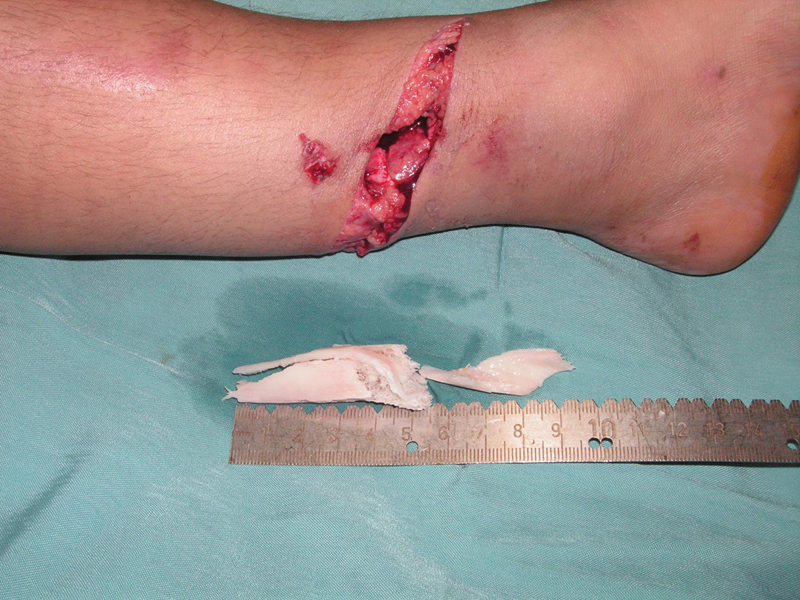
The fragments were prepared for reimplantation.

**Fig. 3:**
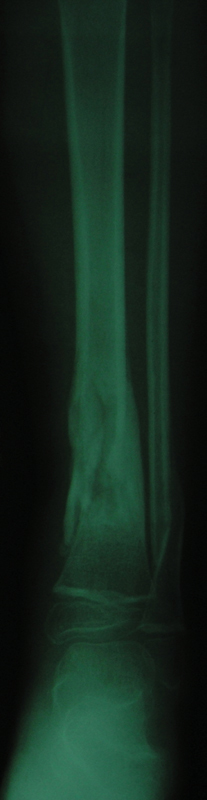
Anteroposterior roentgenogram of the left leg 4 months postinjury demonstrates complete union of the fracture in appropriate alignment. The extruded fragment was incorporated into the callus of the fracture site.

**Fig. 4:**
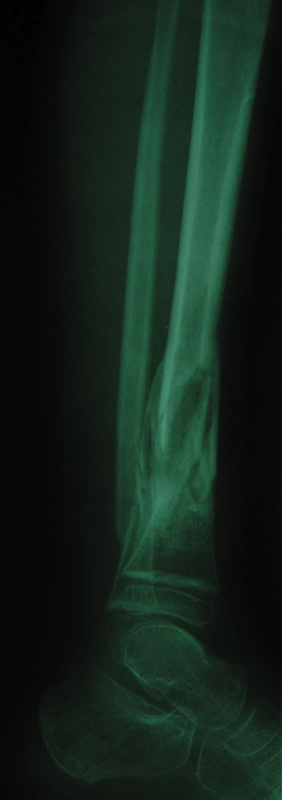
Lateral roentgenogram of the left leg 4 months postinjury demonstrates the fracture in appropriate alignment. The extruded fragment was incorporated into the callus of the fracture site.

The two extruded tibial fragments were cleaned and washed with copious amount of normal saline to remove the gross contaminations. The bone fragments were then scrubbed with 10% povidone–iodine for 20 minutes followed by 20-minute soaking in 2% chlorhexidine gluconate solution. They were then rinsed with normal saline before the reimplantation in the defect area ( Fig. 2 ). The leg was immobilized by a long leg cast, and intravenous cefazolin and gentamicin were administered for 7 days. His postoperative course was uneventful. After 3 months, the patient was able to bear his complete weight and walk without aids, and the knee and ankle joints' range of motions were normal. Figs. 3 and 4 demonstrate complete union of the fracture in appropriate alignment after 4 months. The extruded fragment was incorporated into the callus of the fracture site.

## Discussion

Open fractures are susceptible to infection due to many factors. 3 The extent of soft tissue damage, the way of debridement and irrigation, the duration between trauma and beginning of primary treatment, and sufficient antibiotic coverage are important factors involved in the infection. 3 In addition, in cases with extruded bone segments, factors including adequate sterilization of the extruded fragment, delayed reimplantation, and definitive fixation, as well as the patient's young age and excellent health play a crucial role in the decision regarding reimplantation. 1 2 3

Currently, there is no clear disinfection guideline for traumatically extruded bone segments intended for reimplantation; therefore, we have very limited knowledge. First time in 1965, Kirkup described successful replacement of a 9-inch metadiaphyseal femoral segment after sterilization with boiling and autoclaving. 4 One of the concerns was a higher risk of chronic osteomyelitis after reimplantation. For this reason, the sterilization techniques of the extruded bone segment are of great importance in the final outcome of these patients. Also, Rouvillain et al and Marzurek et al reported similar success in the reimplantation of traumatically extruded metadiaphyseal femoral segments. 5 6 Rouvillain described the technique of sterilization with autoclaving the bone segment at 121°C, 1.3 bars for 20 minutes; then he used the extruded 11-cm metadiaphyseal femur for bone defect filling. 5 Similarly, Marzurek reported chemical sterilization of a 13-cm-long metadiaphyseal femur with chlorhexidine 4% soaking for a total of 270 minutes. 6 Eventually, the duration of the union was similar in both reports, and no complication was observed with full functional recovery. Thermal sterilization is one of the proper techniques, but it can destroy bone osteoinductivity and largely decrease its mechanical strength. 1 2 Another way is to use chemical sterilization techniques. Li et al reported successful reimplantation of an extruded osteoarticular segment of the femur in a rat model with sterilization by povidone–iodine scrub/orthopaedic antibiotic solution. 7 Similarly, Singhi et al described a reimplantation of an extruded metaphyseal segment of the distal femoral condyle following cleaning with copious saline and soaking in 10% povidone–iodine for 20 minutes in a 21-year-old male. 2 In some cases, hybrid sterilization methods have been used. Kumar et al used autoclaving and sterilizing with gentamicin in a reimplantation of a 10-cm-long extruded segment of radius. 8

Aizah et al recently reported the use of irradiation with a dose of 25 kGy on the extruded bone segment in the femoral defect with successful reimplantation for the first time. 1 In our patient, we used chemical sterilization with 10% povidone–iodine for 20 minutes followed by soaking in 2% chlorhexidine gluconate solution for 20 minutes. Based on our experience, this method is simple and accessible and does not affect the bioactivity of the bone. Although many factors are involved in the success of the reimplantation of the extruded segment, the sterilization methods play an important role in patient outcome.

## Conclusion

The preservation of bone biology in an extruded bone segment and its sterilization play a key role in reimplantation success. It seems that chemical sterilization with 10% povidone–iodine for 20 minutes followed by 20-minute soaking in 2% chlorhexidine gluconate solution is a simple and accessible method for sterilization of extruded segment.
